# No effect of 14 day consumption of whole grain diet compared to refined grain diet on antioxidant measures in healthy, young subjects: a pilot study

**DOI:** 10.1186/1475-2891-9-12

**Published:** 2010-03-19

**Authors:** Lynda Enright, Joanne Slavin

**Affiliations:** 1 Department of Food Science and Nutrition, University of Minnesota, 1334 Eckles Avenue, St Paul, MN 55108, USA

## Abstract

**Background:**

Epidemiological evidence supports that a diet high in whole grains is associated with lowered risk of chronic diseases included coronary heart disease, obesity, type 2 diabetes, and some types of cancer. One potential mechanism for the protective properties of whole grains is their antioxidant content. The aim of this study was to compare differences in antioxidant measures when subjects consumed either refined or whole grain diets.

**Methods:**

Twenty healthy subjects took part in a randomized, crossover dietary intervention study. Subjects consumed either a refined grain or whole grain diet for 14 days and then the other diet for the next 14 days. Male subjects consumed 8 servings of grains per day and female subjects consumed 6 servings of grains per day. Blood and urine samples were collected at the end of each diet. Antioxidant measures included oxygen radical absorbance capacity (ORAC) in blood, and isoprostanes and thiobarbituric acid reactive substances (TBARS) in urine.

**Results:**

The whole grain diet was significantly higher in dietary fiber, vitamin B6, folate, selenium, copper, zinc, iron, magnesium and cystine compared to the refined grain diet. Despite high intakes of whole grains, no significant differences were seen in any of the antioxidant measures between the refined and whole grain diets.

**Conclusions:**

No differences in antioxidant measures were found when subjects consumed whole grain diets compared to refined grain diets.

## Introduction

Epidemiological evidence supports that diets high in whole grain foods decrease risk of chronic diseases including coronary heart disease, obesity, type 2 diabetes, and many forms of cancer [1]. Whole grain intake is also linked to biomarkers of disease risk, including an inverse association of whole grain intake to incident hypertension [2]. Unfortunately, in the United States only 1% of individuals consume the recommended three servings of whole grain products per day, and approximately 20% consume virtually no whole grain products [1].

There are several potential protective components in the whole grain that may be lost in the refining process. These include fermentable carbohydrates, phytochemicals, fiber, antioxidants and non-nutrients such as phenolic acids, lignans and phytoestrogens [1]. Components that are found in the outer layers of the whole grain are removed during the milling process. Researchers have focused on a number of these specific components without evaluating their effects as a part of whole foods. Because of the potential synergism between these components in the grain, it is essential to evaluate their effects in whole grain products that individuals are likely to consume.

One of the possible protective mechanisms is the antioxidant capacity of the grain. Antioxidant nutrients found in wheat (one of the largest grain components of diets in the United States) include phenolics, β-carotene, iron, manganese, quercetin, tocopherol, zinc and ascorbic acid [3]. Researchers have evaluated several of these antioxidant nutrients and found them to be protective against certain chronic diseases including coronary artery disease, lung, oral, epithelial and gastric cancers [4]. Dietary studies have also evaluated antioxidant activity *in vivo *after consumption of foods known to be high in antioxidants. Consumption of fruits, vegetables and red wine has been shown to cause an increase in antioxidant activity *in vivo *in young and older adults [5-7]. Research has indicated that whole foods affect antioxidant measures, though little research has been done specifically on the effects of whole grains on antioxidant activity *in vivo*. Because epidemiological research supports that whole grain intake protects against chronic disease and antioxidant research finds that consumption of foods containing antioxidants can increase antioxidant activity *in vivo*, we hypothesized that a diet high in whole grain foods would increase the antioxidant capacity of humans.

## Subjects

Twenty subjects (10 men, 10 women) were recruited from the Minneapolis/St. Paul area by posting flyers. The mean age of subjects was 27.1 ± 4.0 years, mean height 175 ± 10.8 cm, mean weight 73.3 ± 12.6 kg and mean body mass index 23.9 ± 3.3 kg/m^2^. All subjects were non-smokers, with an alcohol intake of less than 2 drinks per day (0.8 oz. ethanol) or less than 5 drinks per week (2.0 oz. ethanol), and a caffeine intake of less than 3 cups or cans of caffeine-containing beverages per day. Subjects had not been on antibiotics in the last 6 months. All subjects consumed low levels (<15 g/day) of total dietary fiber in their regular diet and did not participate in regular strenuous exercise. Subjects were not consuming dietary supplements prior to starting the study and did not consume dietary supplements during the study. Subjects were assessed for exclusion criteria by the investigators during a telephone screening. Subjects received oral and written information about the study and individual written consent was obtained from each subject. All aspects of this research were approved by the University of Minnesota Institutional Review Board Human Subjects Committee. All twenty subjects completed the study.

## Methods

The study was a randomized, crossover design with two 14-day intervention periods with no washout period between treatments. Subjects were assigned to either a diet containing eight servings for men and six servings for women of whole or refined grain foods in addition to their regular diet. After 14 days, they were switched to the other treatment. Subjects were provided with ten food products to choose from, and were instructed to consume the six or eight servings of whole or refined grain foods in addition to their regular diet, while maintaining approximately their regular energy consumption. The foods provided to subjects were commercially available products consisting of cereals, breads, buns, bagels, crackers and cookies. Subjects recorded daily grain product intake on food check-off sheets.

Compliance to the diet intervention was monitored throughout the study by checking subject's diet records and their supplies of grain products. Each subject completed a detailed, estimated diet record for three consecutive days during both the refined and the whole grain diet periods. Nutrient calculations were performed using the Nutrition Data System for Research (NDS-R) software version 4.0, developed by the Nutrition Coordinating Center (NCC), University of Minnesota, Minneapolis, MN, Food and Nutrient Database 28. If an analytical value is not available for a nutrient in a food, NCC calculates the value based on the nutrient content of other nutrients in the same food or on a product ingredient list, or estimates the value based on the nutrient content of similar foods.

All the provided grain products were commercially available and were either whole grain or refined grain. The whole grain cereals met the requirement that 51% of the ingredients were whole grain, while the refined grain cereals included puffed rice and puffed wheat products which were devoid of whole grains. The whole grain breads provided were whole wheat breads while the white, refined, white bread contained no whole grain. Most of the grain products the subject chose to consume daily were cereals and/or breads. Other products provided were chosen to maximize differences in refined and whole grain content. Oatmeal cookies were provided as the whole grain choice while a white flour sugar cookie was the refined grain choice. Cracker choices were saltines as the refined grain choice and a rye crisp as the whole grain choice. Standard serving sizes were used for each food category; for example, one slice of bread, one cookie, 1/2 cup of cereal, etc. The macronutrient composition of the diet treatments is summarized in Table 1. The micronutrient content of the diets is summarized in Tables 2a, b, and 2c. Antioxidant content of a range of plant products including whole grains has been published [8] supporting that commercial whole grain products are concentrated sources of antioxidants.

**Table 1:** Calculated Diet Mean Daily Macronutrient Composition - Diet Treatment

Diet Treatment	Energy (kJ)	Fat (g)	Fat (%)	Protein (g)	Protein (%)	CHO (g)	CHO (%)	Fiber (g)
*Whole Grain*	3090	11	13	22	12	138	75	17

*Refined Grain*	3200	21	25	15	8	129	67	5

**Table 2:** Calculated Diet Mean Daily Micronutrient Composition - Diet Treatment

Diet Treatment	Vit A (μg) RE	β-carot. (μg)	Vit D (mg)	Vit E (mg)	Vit K (μg)	Vit C (mg)
*Whole Grain*	472	6.4	1.3	3.5	16.4	19

*Refined Grain*	55	12.3	.39	2.6	28.6	7

**Diet Treatment**	**Ribofl. (mg)**	**Niacin (mg)**	**Vit B6 (mg)**	**Folate (μg)**	**Vit B12 (μg)**	**Se (μg)**

*Whole Grain*	1.1	14.6	.9	232	.01	66.6

*Refined Grain*	.70	10.9	.5	125	.94	46.9

**Diet Treatment**	**Cu (mg)**	**Zn (mg)**	**Fe (mg)**	**Mg (mg)**	**Met (g)**	**Cys (g)**

*Whole Grain*	.62	5.5	16	152	.38	.49

*Refined Grain*	2.3	1.4	13	57	.27	.31

### Sample Collection

On days 1, 15 and 29 of the study, a fasted 30 ml blood sample was obtained from each subject. Blood samples were collected in EDTA-coated tubes and centrifuged at 1500 × *g *for 15 minutes at 4°C. The plasma layer was removed and stored at -70°C for later analysis. Cell pellets were discarded. Two continuous 24-hour urine samples were collected on days 13 and 14, and days 27 and 28 of the study. Urine samples for each treatment period were pooled for each subject and stored at -20°C until analyzed.

There are numerous methods to evaluate antioxidant activity in foods as well as *in vivo*. Antioxidant content of wholegrain breakfast cereals, Fruits, and vegetables has been published [9]. It is difficult to measure specific antioxidants though it is useful to evaluate both overall antioxidant activity and biomarkers of oxidation. We chose three methods to give a broad picture of antioxidant activity and oxidation in human biological samples.

#### Oxygen Radical Absorbance Capacity

The oxygen radical absorbance capacity (ORAC) assay, developed by Cao et al [9], can be used to measure overall antioxidant capacity of biological fluids. It is believed to be superior to other methods of measuring antioxidant measures because it takes free radical action to completion and combines both inhibition percentage and length of inhibition time of the free radical action by antioxidants into a single quantity [9].

##### Chemicals

Porphyridium cruentum β-phycoerythrin (β-PE) used with the whole plasma samples was purchased from Sigma Chemical (Sigma-Aldrich, St. Louis, MO). Porphyridium cruentum β-phycoerythrin used for the deproteinized plasma samples was purchased from Molecular Probes, Inc. (Eugene, OR). 6-hydroxy 2,5,7,8 tetramethylchroman-2-carboxylic acid (Trolox) was purchased from Aldrich (Milwaukee, WI). 2,2'-azobis (2-amidiniopropane) dihydrochloride (AAPH) was purchased from Wako Chemical USA, Inc. (Richmond, VA). All other chemicals were purchased from Sigma-Aldrich (St. Louis).

##### Sample Preparation

Plasma samples were diluted by mixing 50 μl plasma with 950 μl phosphate buffer to achieve optimum ORAC value. To precipitate the plasma proteins, 100 μl plasma was mixed with 400 μl 100% saturated ammonium sulfate (SAS) and placed in ice water for 20 minutes. The samples were centrifuged at 400 × *g *for 15 minutes at 4°C. The supernatants were removed and stored in ice water for analysis in the same day.

##### Fluorescence Assay

The procedure used is based on the procedure developed by Cao et al. [8]. For this assay, the final reaction mixture of 2 ml contained diluted plasma, plasma fraction or vitamin E analogue Trolox standard, 3.34 × 10^-8 ^M β-PE, 4.0 × 10^-2 ^M AAPH and 7.5 × 10^-2 ^M phosphate buffer, pH 7.0. When whole plasma was assayed, 20 μl phosphate buffer was used as a blank and Trolox was used as a standard. When the deproteinized plasma fraction was assayed, 20 μl 80% saturated ammonium sulfate was used as a blank and Trolox was used as a standard. AAPH was used as the peroxyl radical generator to initiate the reaction and when added the reaction mixture was incubated at 37°C. Fluorescence was measured (Perkin Elmer fluorescence spectrophotometer #650-10S) every one second at the emission of 565 nm and excitation of 540 nm until zero fluorescence of β-PE occurred. This method is based on the loss of fluorescence of β-PE when damaged by oxygen radicals. The ORAC value of each plasma sample was calculated by measuring the net protection area under the quenching curve of β-PE in the presence of an antioxidant. One ORAC unit has been assigned the net protection (S) provided by 1 μM Trolox in final concentration. The ORAC value (units) of the sample is calculated on the basis of a Trolox standard curve as follows:

*k*: dilution factor, S: area under the quenching curve of β-PE (this area is integrated

by a computer connected directly to the output of the fluorescence spectrophotometer).

#### Isoprostane (8-epi prostaglandin F_2α_)

Isoprostanes are prostaglandin-like substances produced during the nonenzymatic, reactive oxygen species dependent peroxidation of lipid-esterified arachidonic acid. They are elevated in conditions of oxidative stress and are therefore a marker of oxidant injury. Research has demonstrated that urinary 8-epi prostaglandin F_2α _is increased in conditions of oxidative stress. As other methods have been criticized for their lack of reliability and accuracy, because this method specifically measures 8-epi prostaglandin F_2a _it is considered a reliable and accurate method to measure oxidative stress *in vivo *[10-13].

##### Sample Preparation

Before analysis, samples were purified by eluting the urine sample through a C18 Sep Pak followed by a Silica Sep Pak. Purified samples were then evaporated under nitrogen gas, and resuspended to a volume of 1 ml in dilution buffer before assaying.

##### ELISA Assay

The isoprostane assay (Oxis International, Inc.) is a competitive enzyme-linked immunoassay kit used to measure free urinary or plasma 8-epi prostaglandin F_2α_. The kit included 8-epi standard, wash buffer, dilution buffer, substrate and 8-epi-hpr-conjugate. The assay is based on 8-epi prostaglandin F_2α _in the sample competing with the 8-epi-hpr-conjugate for binding with horseradish peroxidase. The activity of the enzyme results in color development in the substrate when added. The intensity of the color is proportional to the amount of 8-epi-hpr-conjugate bound and inversely proportional to the amount of 8-epi prostaglandin F_2α _in the samples or standards.

100 μl of standard or sample in duplicate was pipetted into wells. 100 μl of diluted 8-epi-hpr-conjuate was added to each well and incubated for 2 hours in the dark. Following incubation, the plate was washed four times with 400 μl wash buffer using an automated plate washer (El_X_405 BioTek Instruments, Inc.) to remove all unbound reagents and allowed to stand for two minutes between washings. After washing 200 μl substrate was added to each well and incubated for 20 minutes in the dark for development. 50 μl 1 M sulfuric acid was then added to each well and absorbance was measured using an elisa plate reader (El808-IU ultra micro plate reader BioTek Instsruments, Inc.) at 450 nm. The amount of 8-epi prostaglandin F_2α _is expressed in ng per mg creatinine.

#### Thiobarbituric Acid Reactive Substances

The thiobarbituric acid reactive substances (TBARS) assay has been used to evaluate lipid peroxidation and is based on the reaction between malonaldehyde and thiobarbituric acid. The measurement of malonaldehyde in urine is useful because it reflects lipid peroxidation products both from the diet and formed in tissues [14-16]. Since products other than malonaldehyde are known to react with thiobarbituric acid, TBARS are expressed as malonaldehyde equivalents [17,18].

##### Chemicals

Trichloroacetic acid (TCA), thiobarbituric acid (TBA), and malonaldehyde standard were purchased from Sigma Chemical (Sigma-Aldrich, St. Louis, MO). All other chemicals were also purchased from Sigma-Aldrich (St. Louis, MO).

##### Absorbance Assay

Duplicate samples of 0.5 ml urine was combined with 0.5 ml deionized distilled water, 3 ml TCA and 1 ml TBA. Tubes were capped, vortexed and placed in the dark in an 80°C water bath for 90 minutes. At the end of the 90 minute incubation, tubes were again placed in the dark and cooled to room temperature. The absorbance of the urine sample was measured spectrophotometrically (DU Series 500, UV/Vis Spectrophotometer, Beckman Coulter, Inc.) at 535 nm. MDA standards were prepared in the same way as the urine samples. The amounts of TBARS in urine are expressed as ug MDA equivalents per mg creatinine.

### Statistical Methods

There were no other studies of chronic whole grain intake and antioxidant biomarkers when this study was designed, which limited ability to calculate sample size. Cao et al [6] found differences in ORAC when 8 subjects consumed strawberries, spinach, or red wine. Consumption of tomato products significantly decreased isoprostanes when 12 subjects consumed high tomato diets [19]. Also, fruit juice intake increased Trolox-equivalents antioxidant capacity when 5 subjects consumed high juice diets [20]. Thus, twenty subjects were enrolled in this cross-over study.

Data were analyzed by using paired two-tailed student's *t *test between whole grain diet and refined grain diet treatments for diet comparisons and all biomarker activities. Results are expressed as means ± SEMs.

## Results

### Analysis of Subjects' Diets

The macronutrient composition of the subject's habitual diet plus the diet treatment (Table 3) was not significantly different except for a significantly higher quantity of fiber in the whole grain diet. There was some variability however, in micronutrient composition between the diet treatments (Table 4a, b, c). There was a significantly greater content of vitamin B6, folate, selenium, copper, zinc, iron, magnesium and cystine in the whole grain diet treatment when compared to the refined grain diet treatment.

**Table 3:** Calculated Diet Mean Macronutrient Composition - Habitual Diet + Diet Treatment

Diet Treatment	Energy (kJ)	Fat (g)	Fat (%)	Protein (g)	Protein (%)	CHO (g)	CHO (%)	Fiber (g)
*Whole Grain*	9360	72	28	84	15	324	57	26

*Refined Grain*	9470	78	31	81	14	312	55	13

**Table 4:** Calculated Diet Mean Micronutrient Composition - Habitual Diet + Diet Treatment

Diet Treatment	Vit A (μg) RE	β-carot. (μg)	Vit D (mg)	Vit E (mg)	Vit K (μg)	Vit C (mg)
*Whole Grain*	1086	1223	6.3	9.9	53.1	76.6

*Refined Grain*	854	2240	5.9	8.3	74.3	64.2

**Diet Treatment**	**Ribofl. (mg)**	**Niacin (mg)**	**Vit B6 (mg)**	**Folate (μg)**	**Vit B12 (μg)**	**Se (μg)**

*Whole Grain*	2.6	26.7	2.1	351	3.6	149

*Refined Grain*	2.2	25.5	1.7	273	4.7	129

**Diet Treatment**	**Cu (mg)**	**Zn (mg)**	**Fe (mg)**	**Mg (mg)**	**Met (g)**	**Cys (g)**

*Whole Grain*	1.4	13.1	24.4	358	1.8	1.2

*Refined Grain*	1.0	9.4	21.1	253	1.9	1.1

### Analysis of Biological Fluids

#### Subject Exclusions

One subject was not included in any of the analyses of biological fluids because we found that the subject's lipids were not within the range considered normal by the American Heart Association. We felt that elevated plasma lipids may alter antioxidant activity and lipid peroxidation and therefore felt the subject should not be included in analyses. In the isoprostane and TBARS analyses, one additional subject was not included due to incomplete urine collection confirmed by creatinine values. Lastly, a third subject was excluded from the isoprostane analysis due to an elevated coefficient of variation (35% for refined grain diet treatment, 45% for whole grain diet treatment) between duplicate sample analyses which could not be explained. Therefore, the final calculations include 19 subjects in the ORAC results, 17 subjects in the isoprostane results and 18 subjects in the TBARS results.

#### Oxygen Radical Absorbance Capacity

**Figure 1:**
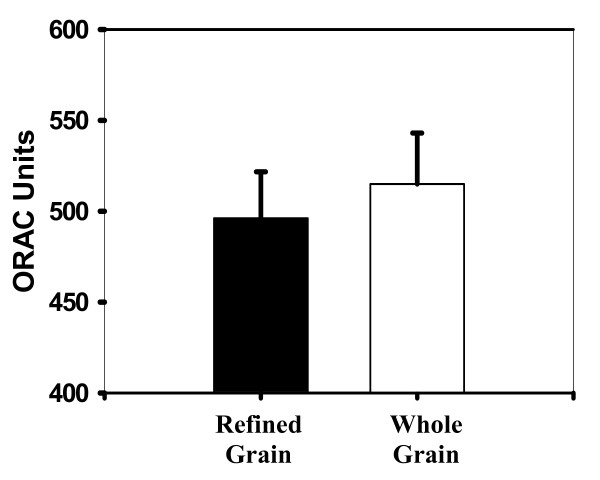
**Oxygen Radical absorbance capacity (ORAC) units of whole plasma samples on refined and whole grain diets**.

**Figure 2:**
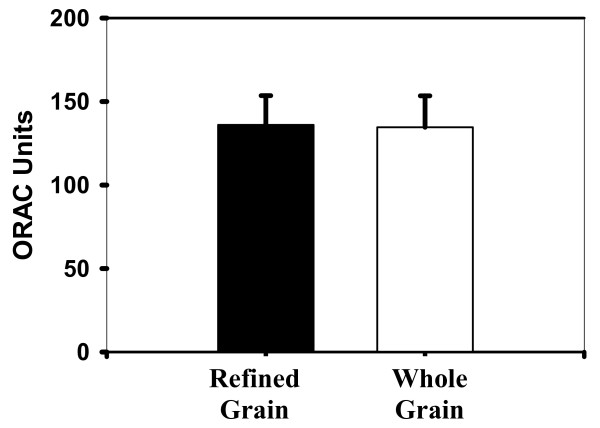
**Oxygen Radical absorbance capacity (ORAC) units of deproteinized plasma samples on refined and whole grain diets**.

The mean antioxidant capacity for the whole plasma is measured in ORAC units, where 1 ORAC unit is equal to the net protection provided by 1 uM Trolox in final concentration. The ORAC units from whole plasma for the refined grain diet and the whole grain diet was 496.12 ± 25.58 ORAC units and 514.96 ± 28.05 ORAC units respectively (Figure 1). The intraassay CV was 8.0% for the whole plasma and 10% for the deproteinized plasma. The mean antioxidant capacity for the deproteinized plasma was 136.05 ± 17.48 ORAC units for the refined grain diet and 134.60 ± 18.80 ORAC units for the whole grain diet (Figure 2). No significant difference was observed between the whole grain and the refined grain diet treatments for either the whole (p = 0.43) or deproteinized plasma portion (p = 0.93).

#### Isoprostane (8-epi-prostaglandin F_2α_)

**Figure 3:**
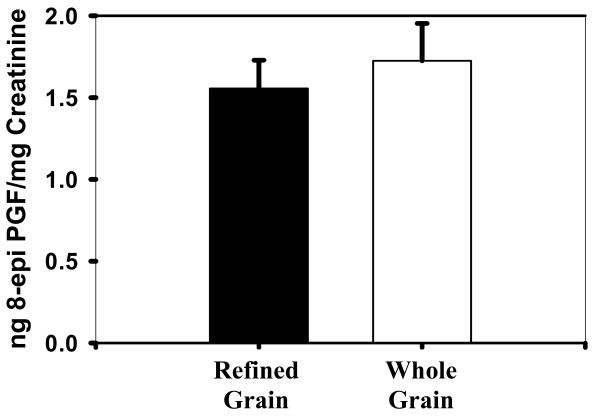
**Urinary isoprostanes on refined and whole grain diets**.

The mean urinary isoprostane was 1.56 ± 0.17 ng 8-epi-prostaglandin F_2α_/mg creatinine for the refined grain diet and 1.72 ± 0.23 ng 8-epi-prostaglandin F_2α_/mg creatinine for the whole grain diet (Figure 3). The intraassay CV was 13%. We expected 8-epi-prostaglandin F_2α _to decrease with the whole grain diet intervention due to the increased antioxidant potential of the diet and the expected decrease in oxidative stress. No significant differences were observed between the whole grain and the refined grain diet treatments for isoprostanes (p = 0.33).

#### Thiobarbituric Acid Reactive Substances

**Figure 4:**
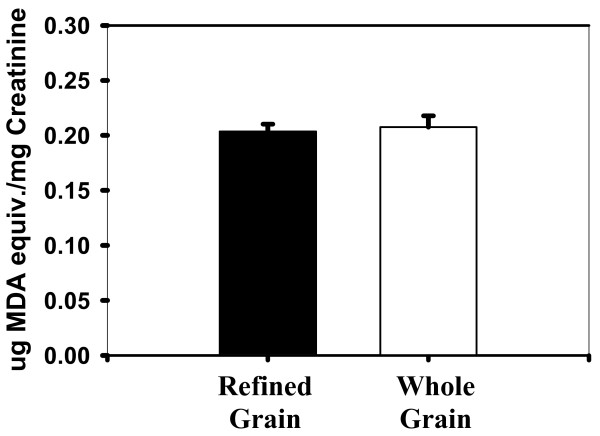
**Urinary thiobarbituric acid reactive substances (TBARS) on refined and whole grain diets**.

Lipid peroxidation *in vivo *measured as mean MDA equivalents was 0.203 ± 0.007 ug MDA equivalents/mg creatinine for the refined grain diet and 0.208 ± 0.010 ug MDA equivalents/mg creatinine for the whole grain diet (Figure 4). The intraassay CV was 15%. TBARS were also expected to decrease with the whole grain diet treatment, again due to a higher antioxidant potential and the expected decrease in oxidative stress. No significant differences were observed between the whole grain and the refined grain diet treatments when evaluating lipid peroxidation using the TBARS method (p = 0.76).

## Discussion

Epidemiological research has shown that whole grains are protective against diseases including cardiovascular disease and many forms of cancer. Previous clinical research has also exhibited a positive association between intake of whole foods, such as fruits, vegetables and red wine, and increased plasma antioxidant capacity [5-7]. However, we found that administration of a diet high in commercially available whole grain foods did not significantly alter the antioxidant activity of healthy adults when compared to a diet high in refined grain foods.

A slight but insignificant increase of approximately 4% was found in the mean antioxidant activity in whole plasma along with a 1% decrease in the deproteinized plasma. These results demonstrate that components in the plasma did not cause a significant delay in the oxidation of the target protein, β-phycoerythrin. In addition, lipid peroxidation increased slightly (by approximately 2%) in evaluation of thiobarbituric acid reactive substances. Also, there was an increase in oxidation of arachidonic acid to form 8-epi-prostaglandin F_2α _with the whole grain diet, though again these differences were not significant. These results revealed that components in the diet did not result in a decrease in lipid peroxidation *in vivo*.

The antioxidant micronutrients, calculated using the nutrition data systems software, were significantly greater in the whole grain diet treatment than the refined grain diet treatment. It is possible that the addition of six or eight servings per day of whole grains may be insufficient to warrant a significant increase in plasma antioxidant activity. However, we believe that this quantity would be sufficient to produce a change in antioxidant activity when compared to the quantities fed in other dietary intervention antioxidant studies [5,21].

An additional factor that may have affected overall antioxidant potential of the diets, in addition to micronutrients calculated with the diet software, is the presence of other antioxidant components such as phenolic compounds (quercetin, caffeic acid or ferulic acid) and synthetic antioxidants (BHT or TBHQ). The presence of phenolic compounds in both diet treatments would have affected overall antioxidant activity, though we expect that these compounds would not be found in significant amounts in refined grain foods because of the nutrient losses that occur in the milling process.

Synthetic antioxidants, however, may more likely have affected antioxidant potential of the diet treatment foods. Several of the products provided to subjects from both diet treatments were known to contain synthetic antioxidants, including BHT and THBQ used for preservation. Research has not yet been conducted to evaluate the antioxidant potential of these synthetic antioxidants in comparison to the "natural" antioxidants found in whole grains. Because we do not know their potential, it is feasible that these antioxidants may have greater antioxidant potential than "natural" antioxidants and may have masked any effect that might have been seen without their presence.

We do believe that the antioxidant nutrients in the whole grain diet were greater than the refined grain diet because of nutrient losses in grain refining. However, it is possible that these nutrients are not being absorbed. The absorption of antioxidant nutrients would be affected by subject characteristics such as age, overall health, or other dietary components, and the bioavailability of these nutrients in the food products provided to subjects. A lack of absorption would explain the fact that no significant change occurred in plasma antioxidants or their protection against lipid peroxidation. Antioxidant micronutrient research has demonstrated an increase in plasma antioxidants with their consumption [22-24]. In addition, whole food research has shown an increase in plasma antioxidants with the consumption of certain fruits and vegetables [5-7,21,25,26]. However, studies have not evaluated the effects of whole grain consumption and antioxidant potential. Therefore, it is not known at this time whether these nutrients are being absorbed in the colon. One way to measure nutrient absorption would be to analyze total antioxidant activity in the food consumed and fecal excretion of antioxidants. Subtracting excretion from consumption would give us a better understanding of nutrient absorption.

The characteristics of the subjects in this study should also be considered in analyzing our results. Physiological stage, aging, and exercise have been shown to be associated with increased oxidation [27,28]. In addition, other factors such as hormonal changes and physical or emotional stress are also believed to be associated with oxidative stress [29]. Because our subjects were healthy, young adults who may not be in a state of excess oxidative stress or antioxidant deficiency, it is possible that a dietary intervention such as this may not have warranted a change in antioxidant protection.

One of the main objectives of this study was to evaluate the effects of a whole grain diet in a free-living situation in the average individual. Controlling subjects' consumption and lifestyle may have provided more easily measured dietary antioxidant potential, though would not have provided practical information that could be applied to the average individual. Also choosing a population in a state of known oxidative stress might have provided a situation in which a change was more visible. However, this would not answer the question of whether or not a dietary intervention that is practical for the average individual would be effective in increasing antioxidant potential *in vivo*.

Other whole grain feeding studies have found few changes in biomarkers. While whole-grain wheat breakfast cereal had a prebiotic effect in human subjects, there were no differences blood glucose, insulin, or blood lipids with whole grain consumption compared to refined grain consumption [30]. Also, whole-grain foods did not affect insulin sensitivity or markers of lipids peroxidation and inflammation in health, moderately overweight subjects [31].

It is possible that the diet composition or subject characteristics were not ideal for significant alteration of antioxidant activity in our study. However, we believe that it is more likely that the mechanism by which the protection of whole grains occurs is not due solely to its antioxidant potential but to some of the other components of the whole grain, or a combination of many of its components.

## Competing interests

The authors declare that they have no competing interests.

## Authors' contributions

LE carried out the intervention trial, wrote the manuscript, and completed the biological measures. JS designed the study, oversaw the operation of the study, assisted in the interpretation of results, and revised the manuscript. Both authors read and approved the final manuscript.
